# Antiviral effect of baicalin on Marek’s disease virus in CEF cells

**DOI:** 10.1186/s12917-020-02595-x

**Published:** 2020-10-02

**Authors:** Fan Yang, Chun Feng, Yongxiu Yao, Aijian Qin, Hongxia Shao, Kun Qian

**Affiliations:** 1 Ministry of Education Key Lab for Avian Preventive Medicine, Yangzhou University, No.48 East Wenhui Road, Yangzhou, Jiangsu 225009 P.R. China; 2 Jiangsu Key Lab of Preventive Veterinary Medicine, Yangzhou University, No.48 East Wenhui Road, Yangzhou, Jiangsu 225009 P.R. China; 3 The International Joint Laboratory for Cooperation in Agriculture and Agricultural Product Safety, Ministry of Education, Yangzhou University, Yangzhou, 225009 P.R. China; 4 The Pirbright Institute & UK-China Centre of Excellence for Research on Avian Diseases, Pirbright, Surrey GU24 0NF UK; 5 Institute of Comparative Medicine, Yangzhou University, Yangzhou, Jiangsu 225009 P.R. China; 6 Yangzhou, P. R. China

**Keywords:** Marek’s disease virus, Baicalin, Antiviral activity, Inhibition

## Abstract

**Background:**

Baicalin, the main metabolic component of *Scutellaria baicalensis* Georgi, has various pharmacological properties including anti-inflammatory, anti-oxidant, anti-apoptotic, anti-bactericidal and anti-viral. The purpose of this study was to investigate the anti-Marek’s disease virus (MDV) activities of baicalin in CEF cells.

**Results:**

Here, we showed that baicalin could inhibit viral mRNA, protein levels and overall plaque formation in a time-dependent manner. We also found that baicalin could consistently inhibit MDV replication and directly affect the virus infectivity. Moreover, baicalin treatment has no effect on expression level of antiviral cytokine and inflammatory cytokines in MDV infected CEFs.

**Conclusions:**

These results demonstrate that baicalin could be a potential drug against MDV infection.

## Background

Marek’s disease virus (MDV) is a member of *alpha*-herpes virus subfamily, and serotype 1 strains are the etiologic agentof Marek’s disease (MD), which is a highly contagious and infectious malignant lymphoid neoplastic disease. MD is a neoplastic disease of chickens and other gallinaceous birds. It is one of the major diseases that threatens poultry industry worldwide and causes heavy economic losses [1]. Current report showed that half of the world countries had case reports of MDV infection [2]. In recent years, many reports of the onset of MDV have also been declared in vaccinated chickens [3–6]. The virulence of MDV has been continuously evolved and gradually increased under the pressure of vaccination. In MD vaccine can prevent tumorigenesis and immunosuppression, but the virus can still replicate into fully infectious virus particles and shed cell free mature virions through skin dander and dust. Thus, the ideal MDV vaccine would be capable of controlling the replication of MDV in chickens and stopping the shedding of virions [2].

Traditional Chinese Medicine (TCM) has been widely used to treat and prevent illnesses for thousands of years. Baicalin, a flavonoid compound, is the main effective component of *Scutellaria baicalensis* Georgi which is one of the commonly used herbal medicine in China [7]. Previous studies have reported that baicalin exibited inhibitory effects against Herpes simplex virus 1 (HSV-1) [8]. It could inactivate free Dengue virus (DENV) particles and interfere with intracellular viral replication by affecting the attachment of DENV to host cells [9]. As a neuraminidase (NA) inhibitor, it had anti-influenza A virus infection activity [10]. It could also directly kill Newcastle disease virus (NDV) and block intracellular NDV replication. In our previous report, we showed that baicalin had antiviral effect on avian leukosis virus subgroup J through targeting virus internalization [11]. However, the inhibitory effects of baicalin on MDV infected CEF cells were not evaluated. Therefore, the present study focused on the antiviral properties of baicalin and its mechanism against MDV replication in CEFs.

## Results

### Cytotoxicity of baicalin on CEF cells

**Fig. 1:**
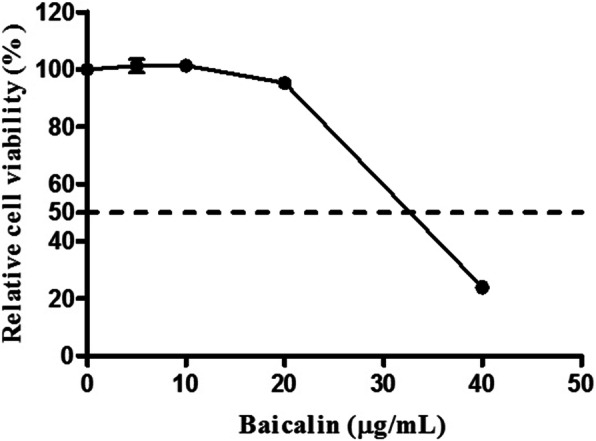
Cell viability of CEF cells with various concentrations of baicalin. The cells were treated with a series of concentrations (0, 5, 10, 20 and 40 μg/mL) of baicalin for 72 h. The relative cell viability was calculated as (OD_450_ drug)/(OD_450_ control) × 100%. The dotted line shows the 50% cell viability position. Data are expressed as the mean ± S.D. of three independent experiments

In order to exclude the possibility that the antiviral activity was due to cytotoxicity of the chemical, a CCK-8 cytotoxicity assay was performed. After treatment with baicalin at concentrations of 5, 10 and 20 μg/mL, the relative cell viability was above 90%, whereas the viability was under 50% after treatment with baicalin at the concentrations of 40 μg/mL (Fig. 1) No difference in cell morphology was observed at a concentration of ≤20 μg/mL. Thus, baicalin was used at a concentration of no more than 20 μg/mL in the subsequent experiments.

### Antiviral activity of baicalin on replication of MDV in CEF cells

**Fig. 2:**
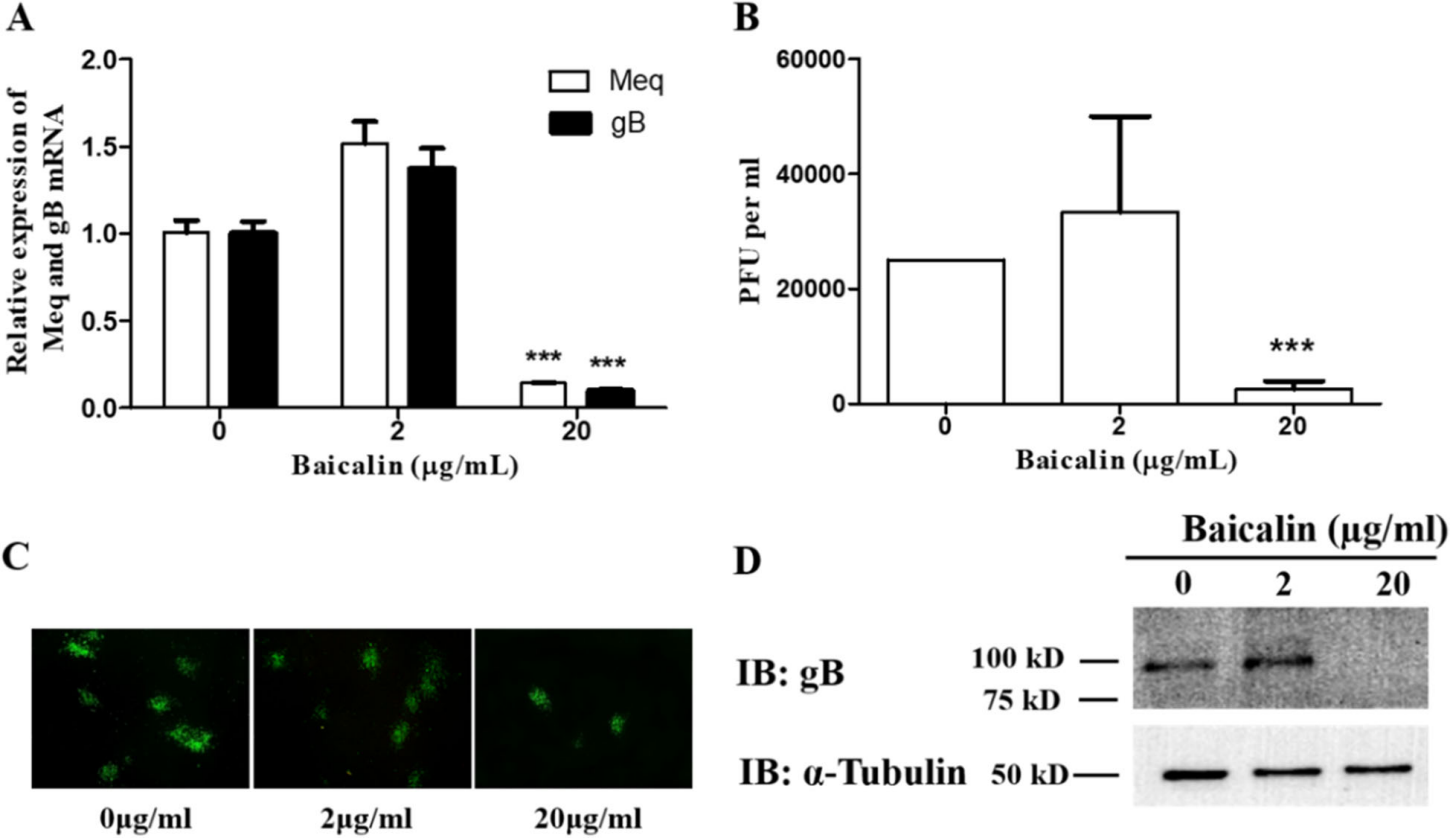
Baicalin inhibits the replication of MDV in CEF. **a** The expression levels of Meq and gB genes in RB-1B strain of MDV infected CEF were quantified by qRT-PCR after treatment with 0, 2 and 20 μg/mL baicalin. **b** The result of plaque count of RB-1B infected CEF treated with 0, 2 and 20 μg/mL of baicalin. **c** Inhibitory effects of plaque formation of MDV RB-1B in CEFs by indirect immunofluorescence assays. **d** The viral encoded gB protein levels were measured by immunoblotting at 96 h p.i. post baicalin treatment. The α-tubulin was used as loading control. The full-length blots are presented in Supplementary Figure 1. Data were expressed as mean ± SD from three independent experiments and analyzed by Student’s t-tests (*** *p* < 0.001)

After 96 h of inoculation, it was found that the transcription levels of the Meq and gB genes were significantly decreased at concentration of 20 μg/mL compared with the control group (Fig. 2a). The results of viral plaque counting, indirect immunofluorescence staining, and Western blot analysis also showed that baicalin at 20 μg/mL significantly inhibited viral protein expression and plaque formation (Fig. 2 B-D). In contrast, the 2 μg/mL low-dosage group did not show any significant inhibitory effect. These results indicate that 20 μg/mL baicalin has significant antiviral activity against MDV RB-1B infection in CEF cells.

### Time-dependent manner of baicalin inhibition on MDV replication

**Fig. 3:**
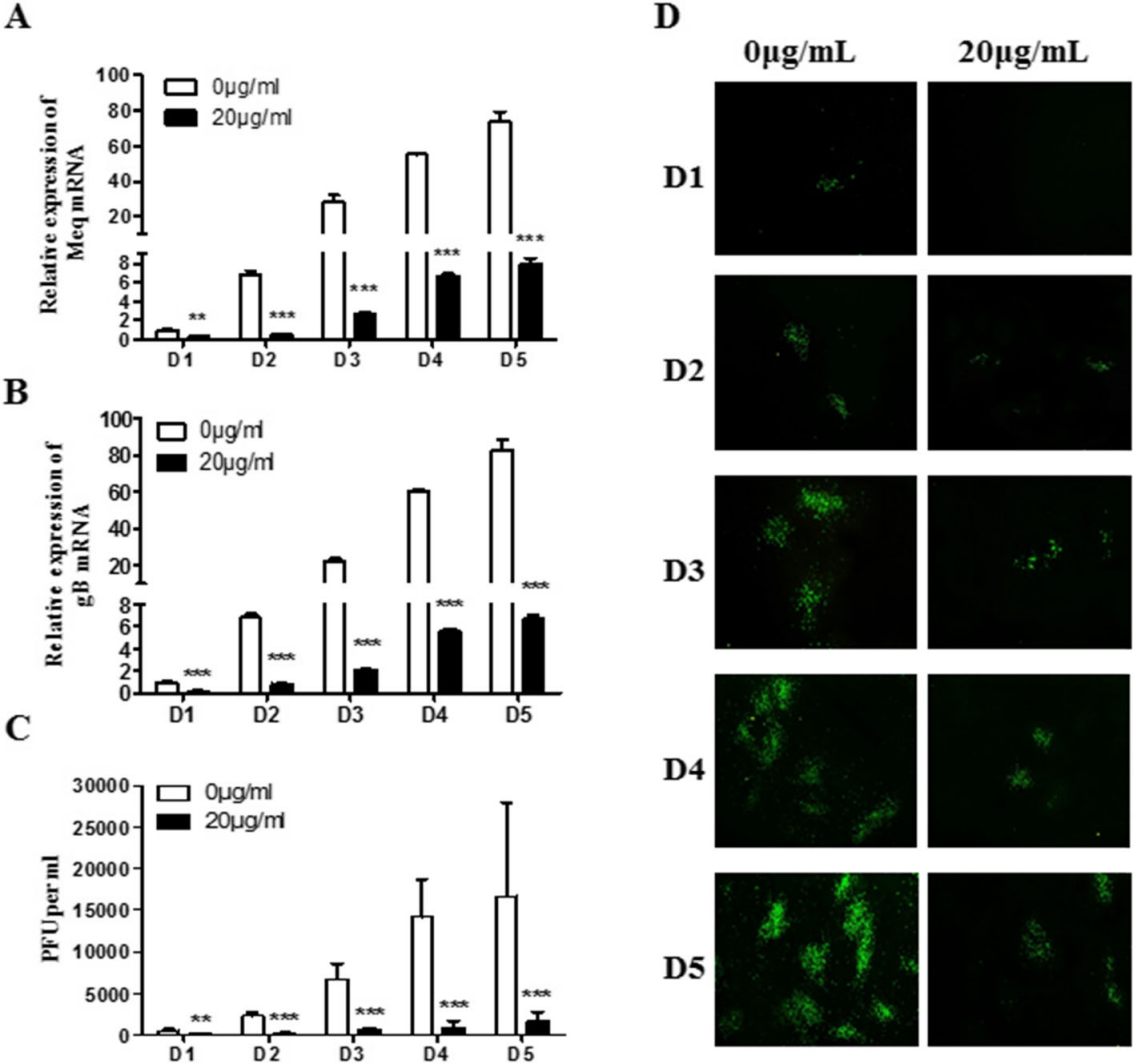
Time-dependent manner of baicalin inhibition on MDV replication in CEF. Total cellular RNA was extracted from day 1 to day 5 p.i., and the expression levels of Meq gene (**a**) and gB gene (**b**) were detected by qRT-PCR. **c** The results of plaque count of the MDV RB-1B strain infected CEF treated with 0 and 20 μg/mL of baicalin at different time points. **d** Direct observation of viral plaque formation dynamics by indirect immunofluorescence assays from day 1 to day 5 post virus infection. Data were expressed as mean ± SD is for A, B and C only from three independent experiments and analyzed by Student’s t-tests (**p < 0.01, *** *p* < 0.001)

Virus-infected CEF cells were harvested at 24, 48, 72, 96, and 120 h after viral infection for plaque counting, real-time PCR and indirect immunofluorescence assay. The viral gene expression was continuously inhibited at different time points after treatment with baicalin at 20 μg/mL. Significant differences were observed from D1 to D5 post-infection as shown in Fig. 3a, b, and this inhibitory effect is alleviated when viral infection level increases. Viral plaque counting and indirect immunofluorescence assay results were also consistent with real-time PCR results (Fig. 3 C and D). Overall these results suggest that baicalin at 20 μg/mL had a significant inhibitory effect on MDV replication in a time-dependent manner.

### Inhibitory effects of baicalin depending on the time of application

**Fig. 4:**
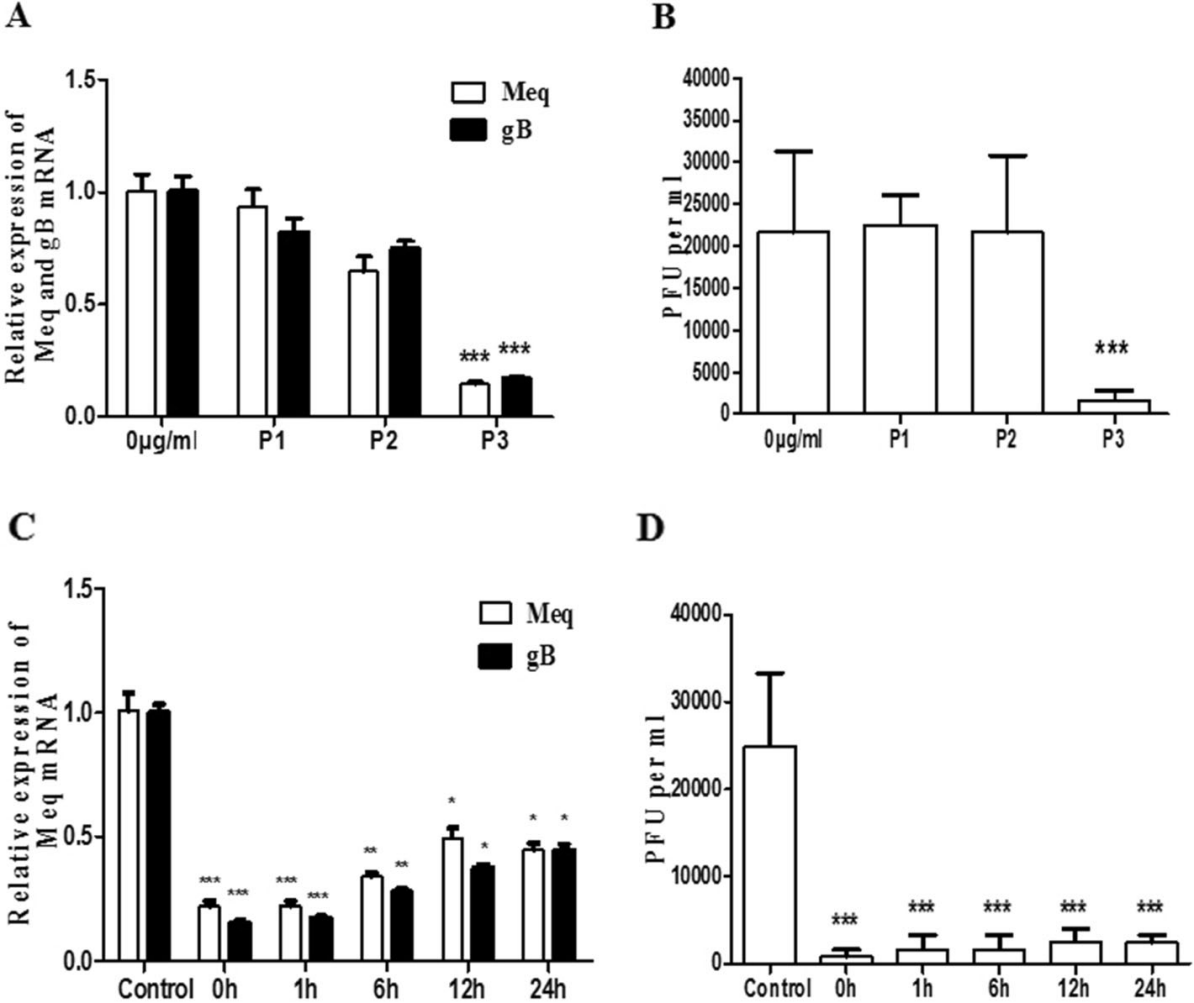
Effects of baicalin on MDV RB-1B replication are dependent on the time of baicalin application. **a** The gene expression level of Meq gene and gB gene were detected by qRT-PCR **b** Viral quantity was detected by plaque counting. 0 μg/mL indicates no baicalin treatment, P1 indicates pretreatment with 20 μg/mL baicalin for 2 h before virus adsorption, P2 indicates 20 μg/mL baicalin and the virus were added at the same time, and P3 indicates 20 μg/mL baicalin was added after virus adsorption. (C + D) 20 μg/mL baicalin was added at 0, 1, 6, 12, and 24 h after virus adsorption, and the virus was harvested 96 h post infection for detection of Meq and gB expression by qRT-PCR (**c**), and virus quantification by plaque counting (**d**). Data were expressed as mean ± SD from three independent experiments and analyzed by Student’s t-tests (*p < 0.05,**p < 0.01, *** *p* < 0.001)

According to the experimental design, three time points of drug application were carried out. Real-time PCR and plaque counting experiments revealed that baicalin had significant inhibition on MDV replication only in the P3 protocol, which 20 μg/mL of baicalin was added to maintainance medium after virus adsorbing. But significant inhibition was not observed in the P1 and P2 protocol (Fig. 4 A, 4B).

In order to investigate the time points exerting the antiviral effect of baicalin on the viral intracellular replication cycle, baicalin (20 μg/mL) was added to CEF cells at 0, 1, 6, 12, and 24 h after virus adsorption, respectively. The results in Fig. 4c and d demonstrated that at the level of viral gene expression, the inhibitory effect weakened over time, but the persistence of viral plaque formation was significantly inhibited by baicalin.

### Baicalin directly affects infectivity of MDV

**Fig. 5:**
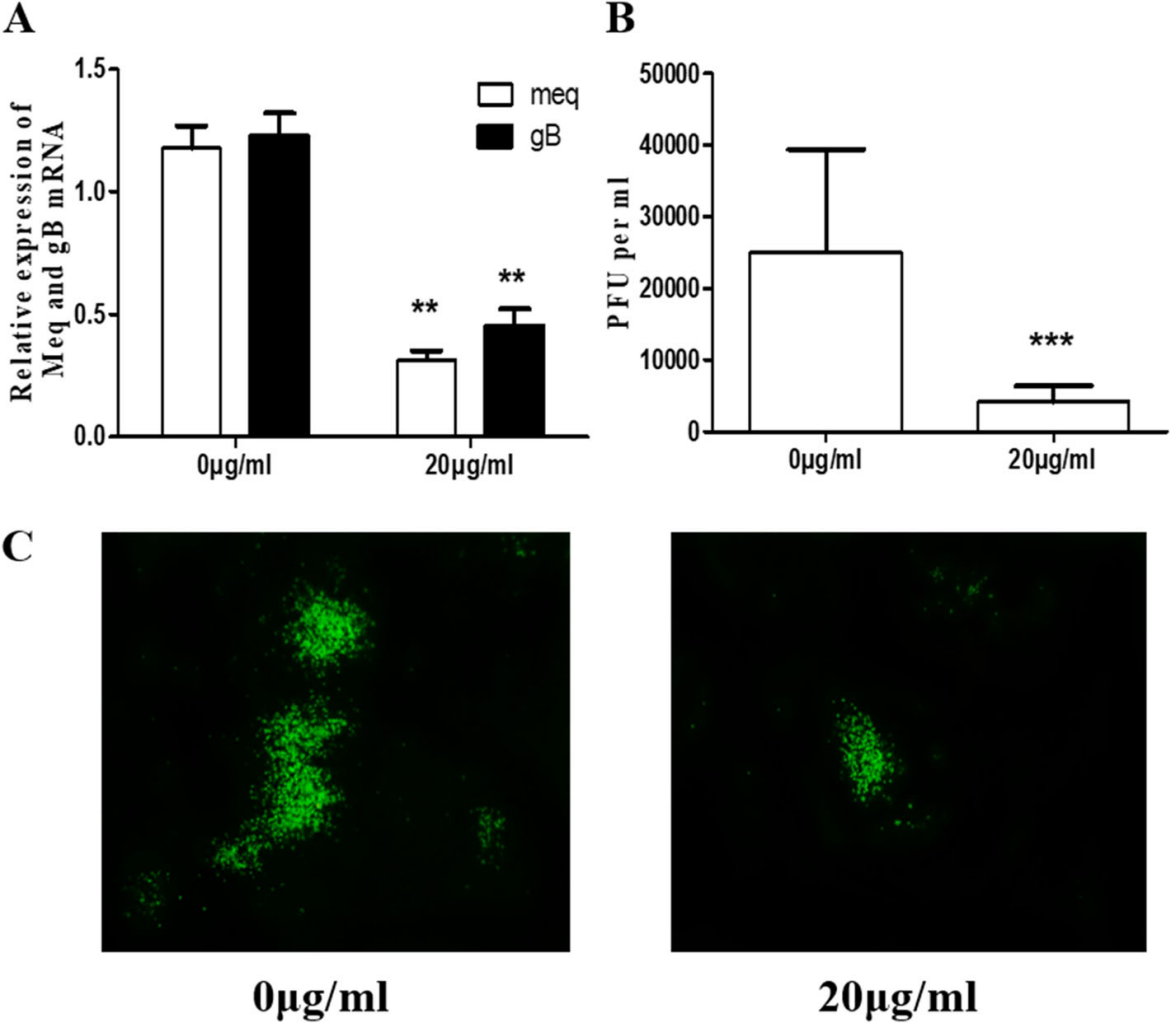
Direct virucidal activity of baicalin on MDV. Compounds were mixed with MDV at 0 and 20 μg/mL respectively and incubated in a 37 °C incubator for 1.5 h, then inoculated onto CEF until harvest. The RNA of the cells were extracted for qRT-PCR to detect the expression level of the Meq gene and the gB gene (**a**). The virus quantity was detected by plaque counting (**b**). The indirect immunofluorescence assay showed the similar results with plaque counting (**c**). Data were expressed as mean ± SD from three independent experiments and analyzed by Student’s t-tests (**p < 0.01, *** *p* < 0.001)

The virus suspension was incubated with the drug for 1.5 h at 37 °C, and CEF cells were infected for 96 h. Real-time PCR and plaque counting results showed that viral gene expression and virus replication were significantly lower in the drug-treated group compared to untreated virus suspension group (Fig. 5). This result indicates that 20 μg/mL baicalin can directly inhibit the infectivity of MDV.

### No effect of Baicalin on pro-inflammatory cytokines and IFN-β gene expression in MDV infected CEF cells

**Fig. 6:**
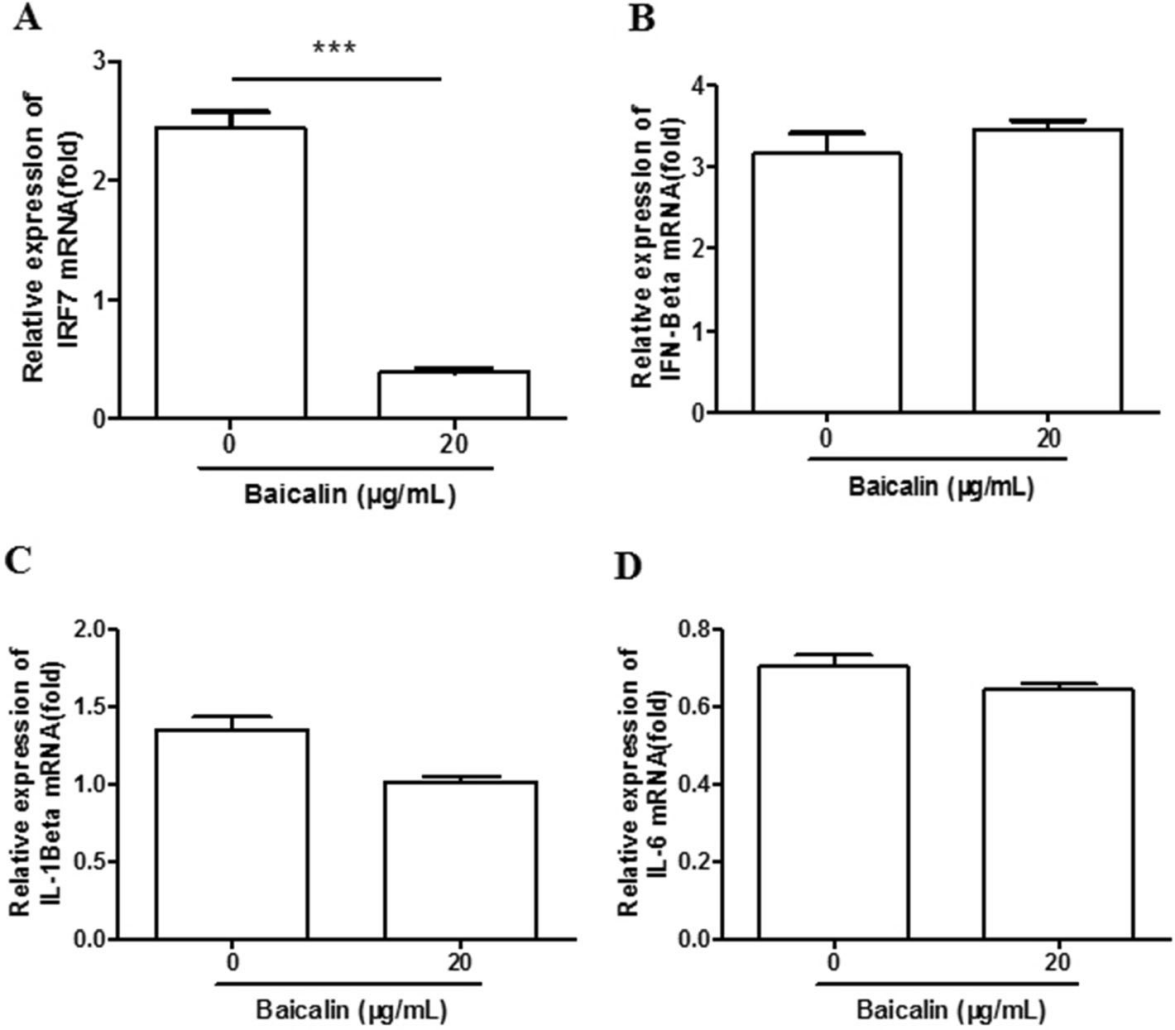
The effect of baicalin on the expression of IFN regulatory factor and cytokines. **a** The expression of IFN regulatory factor IRF7 after baicalin treatment and MDV infection. **b** The expression of IFN-β after baicalin treatment and MDV infection. The expression of proinflammatory cytokines IL-1β (**c**) and IL-6 (**d**) after baicalin treatment and MDV infection. Data were expressed as mean ± SD from three independent experiments and analyzed by Student’s t-tests (*** *p* < 0.001)

Anti-inflammatory effects of baicalin in vitro and in vivo have been reported before [12, 13]. In this study, we investigated whether baicalin affected the expression of pro-inflammatory cytokines and IFN-β in virus-infected CEF cells. The results in Fig. 6 showed that baicalin significantly reduced the mRNA levels of IFN regulatory factor IRF7, whereas no significant difference was observed at the mRNA levels of IL-6, IL-1β and IFN-β.

## Discussion

It is well known that MDV vaccine can provide protective immunity but not sterile immunity which means that the vaccine can inhibit the development of lymphoma but can not prevent virus replication and shedding [2]. Therefore, the development of effective antiviral drugs has positive significance for the prevention and control of MD.

In recent years, the research on antiviral property of Traditional Chinese Medicine has attracted the attention of research groups worldwide due to its minimal side effect and low toxicity [14]. The baicalin as a flavonoid is the metabolite of baicalein and the evidence of its antiviral role against HSV-1, Herpes simplex virus 2 (HSV-2), Zika virus (ZIKV), Influenza A virus (IAV), Duck hepatitis A virus type 1 (DHAV-1), and NDV has been reported [7, 8, 15–17]. In our previous study, we demonstrated that baicalin has an specific inhibitory effect on ALV-J infection by reducing the infectivity and affecting virus internalization [11]. In the current study, we showed that baicalin exhibited significant inhibitory activity against MDV infection in CEFs in a time-dependent manner, with decreased viral gene and viral protein expression as well as plaque counts (Fig. 1 and Fig. 2). This is similar to the results of previous studies on antiherpetic activities of flavonoids against herpes simplex virus [8]. To explore the antiviral mechanism, the drug was added at different time points and virucidal assay were carried out. The results showed that baicalin could consistently inhibit MDV replication in the viral replication cycle when baicalin was added post infection in CEFs, while no antiviral effect was observed in the pre-treatment and virus absorption steps (Fig. 4). In addition, the plaque formation and yield were reduced significantly in virucidal assay (Fig. 5), which was consistent with previous reports that other chemical compounds extracted from Chinese medicinal herbs such as dipotassium glycyrrhizinate and sodium tanshinone IIA sulfonate could directly inactivate MDV particles also [18]. This result suggests that different TCM from different chemical family may share the same antiviral mechanism. Published study has shown that baicalin can inhibit the NDV infection by blocking HN binding to sialic acid-containing receptors and virus adsorption [15], however we do not know how baicalin affects the spread of MDV from cell to cell yet as the mechanism of how MDV enters the host cells is not fully understood.

In MDV infected CEF cells, the expression level of IFN regulatory factor 7 (IRF7) increased obviously [19]. After baicalin treatment, the IRF7 expression decreased significantly, which was synchronized with viral replication reduction. It is well known that IRF7 is an important transcription factor of interferon. However, the expression of IFN-β did not change despite of the significant down-regulation of IRF7 with baicalin treatment in this study (Fig. 6b), suggesting that the expression of IFN-β caused by MDV infection is regulated by other transcription factors. In addition, the expression levels of pro-inflammatory factors IL-1β and IL-6 were slightly decreased in MDV infected CEF cells treated with baicalin, but the difference was not significant. This is inconsistent with previous study that baicalin controls influenza A virus infection by down-regulating the RIG-I-like receptors signaling pathway [20]. This difference may be related to the different antiviral mechanisms of baicalin in different cells.

## Conclusions

In conclusion, baicalin displayed strong anti-MDV effect in a time dependent manner. Furthermore, baicalin reduces MDV replication after viral adsorption and affects the virus infectivity when MDV was pre-incubated with baicalin before infecting CEFs. Moreover, baicalin reduced the expression level of IRF7, but had no effect on expression of inflammatory cytokines in virus infected CEFs. Taken together, these results suggest that baicalin has potential to be further developed as anti-MDV drugs for use in clinic. Future studies will explore the in vivo antiviral effects of baicalin in chickens.

## Methods

### Virus, cells and reagents

The RB-1B strain of MDV is cell-associated virus, and it was stored in our laboratory in liquid nitrogen. Chicken embryo fibroblast (CEF) cells were prepared from 9 days old SPF chicken embryo. The SPF chicken embryos were obtained from the Merial Vital Laboratory Animal Technology Co., Ltd. (Beijing, China), and the animal experiments were performed according to the institutional animal care guidelines and approved by the Animal Care Committee of College of Veterinary Medicine, Yangzhou University. The fully confluent monolayers of CEF cells were grown in Dulbecco’s modified Eagle medium (DMEM; GIBCO, Shanghai China) supplemented with 5% fetal bovine serum (FBS), 100 U/mL of penicillin, and 100 g/mL of streptomycin at 37 °C in a 5% CO_2_ atmosphere. Baicalin was purchased from SIGMA (Shanghai, China) and diluted in dimethyl sulphoxide (DMSO). The specific monoclonal antibody against MDV gB protein, BA4, was generated in our laboratory. And the anti-tubulin monoclonal antibody was purchased from SIGMA (Shanghai, China).

### Cytotoxicity test

The cytotoxicity of baicalin on CEF cells was determined using a cell counting kit, CCK-8 kit (Beyotime, Shanghai, China). Briefly, 4.0 × 10^4^/well CEF cells were seeded in 96-well plates, and 150 μL of baicalin at 0, 5, 10, 20 and 40 μg/mL in DMEM maintenance medium was added to each well. After incubating for 72 h in a 37 °C incubator, CCK-8 solution (15 μL) was added to each well. Incubation was continued for 1 h and the absorbance at 450 nm was measured. The relative cell viability rate was determined as a percentage for each concentration as (OD_450_ drug)/(OD_450_ control) × 100.

### Virus infection and chemical treatment

To investigate the inhibitory effect of baicalin on MDV replication, CEF cells were seeded in 12-well plates (6.0 × 10^5^ cells/well) and pretreated for 2 h at 37 °C with DMEM maintenance medium containing 0.5% serum and 0, 2 or 20 μg/mL baicalin. The cells were then infected with 200 PFU of MDV and incubated under the exposure of the drug for 96 h before harvesting. The collected cells were used for plaque counting, real-time PCR, western blotting and indirect immunofluorescence assay.

To determine the dynamics of baicalin inhibition of viral replication, CEF cells in 12 well plates were pretreated with 20 μg/mL baicalin for 2 h, and then 200 PFU of MDV per well was inoculated. After 4 h of virus adsorption, the medium was replaced by 20 μg/mL baicalin diluted in DMEM maintenance solution with 0.5% serum. Virus infected cells were harvested at 1, 2, 3, 4 and 5 days respectively for plaque counting, real-time PCR detection and indirect immunofluorescence assay.

### Different models of drug treatment and virus infection

In order to investigate the mechanism of viral inhibition by baicalin, a time-of-drug addition experiment was carried out as described previously with minor modifications [11]. Briefly, in the first protocol (P1), CEF cells were pre-treated with 20 μg/mL baicalin for 2 h at 37 °C before viral adsorption. After removing the culture medium containing the drug, the cells were inoculated with 200 pfu virus and incubated with the maintainance medium containing 0.5% FBS at 37 °C in an atmosphere of 5% CO_2_ until harvest; in the second protocol (P2), a mixture of 200 PFU virus suspension and 20 μg/mL of baicalin were added to cell monolayers and incubated for 2 h at 37 °C. After removing the medium, the cells were incubated with maintainance medium at 37 °C in an atmosphere of 5% CO_2_; in the third protocol (P3), 20 μg/mL of baicalin was added to maintainance medium after virus adsorbing for 4 h in a 37 °C incubator. After 96 h of incubation, viral gene expression and plaque formation were examined for all protocols. In order to understand the correlation between baicalin and virus infection, the time course analysis was performed. CEF cells were infected with 200 PFU MDV for viral adsorption. Baicalin (20 μg/mL) was then added into the cells at 0 h, 1 h, 6 h, 12 h, and 24 h after virus adsorption, respectively. At 96 h post infection (p.i.), cells were haversted for viral gene expression and plaque formation.

### Virucidal assay

Two hundred pfu virus were mixed with 20 μg/mL of baicalin and incubated at 37 °C for 1.5 h. After centrifugation to remove the supernatant, the virus-infected cells were resuspended and added to CEF in a 12-well plate. The inoculum was replaced with fresh 0.5% serum containing maintenance medium after 4 h of virus adsorption. The cells were harvested after 96 h post infection for plaque counting and real-time PCR.

### Plaque counting

Virus infected CEF cells in 12-well plate were digested with 400 μL 0.05% trypsin per well. 100 μL per well of 10 fold serial diluted cells were added into 96-well CEF cells with 12 replicates of each dilution The cells were incubated at 37 °C in an atmosphere of 5% CO_2._ The number of viral plaques was counted after 96 h. The highest dilution which has plaques in all 12 wells was used for titer calculation. The formula used is PFU/mL = (total plaques/12) × 10 × dilution factor.

### Quantitative reverse transcriptase polymerase chain reaction

The expression levels of the viral genes and cytokines were determined with real-time PCR (7500 Real-Time PCR System, ABI) as previously reported [21]. The sequences of the primers are provided in Table 1, and these primers were synthesized by Gene Script Company (Nanjing, China).

**Table 1:** Primers used for real-time PCR

Target gene	Sequence	Product size	Accession number
Meq	F 5′-GTCCCCCCTCGATCTTTCTC-3′ R 5′-CGTCTGCTTCCTGCGTCTTC-3’	184	NC-002229.3
gB	F 5’-ACCCCATTCGGTGGCTTTTC-3′ R 5′-GCGTCCAGTTGTCTGAGG-3’	122	NC-002229.3
IRF7	F 5’-CGTATCTTCCGCATCCCTTGG-3’	206	NM-205372.1
	R 5’-TCGTCGTTGCACTTGGAGCG-3’		
IFN-β	F 5’-GCTCTCACCACCACCTTCTC-3′ R 5′-GCTTGCTTCTTGTCCTTGCT-3’	151	NM-001024836.1
IL-1β	F 5’-TAGATGTCGTGTGTGATGAG-3′ R 5′-GTAGAAGATGAAGCGGGTC-3’	105	NM-204524.1
IL-6	F 5’-CAGGACGAGATGTGCAAGAA-3′ R 5′-TAGCACAGAGACTCGACGTT-3’	233	NM-204628.1
18S	F 5′-TCAGATACCGTCGTAGTTCC-3’	154	AF173612
	R 5′-TTCCGTCAATTCCTTTAAGTT-3’		

Total RNA from CEF cells was prepared using the AxyPrep Multisource Total RNA Miniprep Kit (AXYGEN, USA). 1 μg of RNA was reverse transcribed into cDNA using PrimeScript RT Master Mix (TaKaRa, USA). The expression levels of viral genes were determined by real-time SYBR green quantitative PCR (7500 Real-Time PCR System, ABI). The diluted cDNA (1 μL), 400 nM primer and 10 μL of SYBR Green Master Mix were used for real-time PCR in a total volume of 20 μL reaction. The amplification conditions were as follows: 95 °C for 30 s, then 40 cycles, 95 °C for 5 s and 60 °C for 34 s. A dissociation curve was generated to analyze each PCR product after 40 cycles. The analysis of relative gene expression data was performed using the 2^-ΔΔCT^ method with the chicken 18S as the internal reference gene.

### Indirect immunofluorescence

The infected cells were fixed with acetone ethanol (3:2) for 7 min at room temperature. After washing three times with PBS, the fixed cells were incubated with anti-gB mAb BA4 (5 μg/mL) in PBS for 60 min at 37 °C, then washed with PBS followed by incubation with goat anti-mouse IgG conjugated with FITC (Sigma, USA) at room temperature for an additional 30 min. The pictures were captured with a OLYMPUS fluorescence microscope.

### Western blot analysis

Similar to our previous report [11], after cell lysing with RIPA buffer, the protein concentration was determined using a BCA Protein Assay Kit (Bio-Rad, USA). The proteins (30 μg) were denatured by heating (5 min, 100 °C) and electrophoretically separated in 12% SDS-PAGE under reduction conditions. The protein was then transferred onto a nitrocellulose membrane (Sigma, Shanghai, China). The membrane was blocked with 5% skimmed milk containing PBST (PBS containing 0.1% Tween 20) for 1 h and incubated with the anti-gB monoclonal antibody BA4 (10 μg/mL) and anti-tubulin antibody (10 μg/mL) as internal control for 2 h at room temperature. The membrane was washed three times with PBST and incubated with the appropriate HRP secondary antibody (SIGMA, Shanghai, China) for 60 min at 37 °C. After washing with PBST, the membrane was developed using Bio-Rad enhanced chemiluminescent substrate and photographed with an ultrasensitive chemiluminescence detector (Proteinsimple, USA).

### Statistical analyses

The results represent the means ± standard deviations (SD) of triplicate determinations. The significance of the variability between the trials was analyzed using GraphPad (version 5.0) software. Differences between samples were assessed by the Student’s t-test, and *p* values < 0.05 were statistically significant. The experiment was performed at least three times independently with the similar results.

## Supplementary information

**Additional file 1 Figure S1.** The full-length blot images of Fig.2d in the manuscript.

## Data Availability

The datasets used and/or analysed during the current study are available from the corresponding author on reasonable request.

## Abbreviations

MD: Marek’s disease
MDV: Marek’s disease virus
ALV-J: Avian leukosis virus subgroup J
NDV: Newcastle disease virus
TCM: Traditional Chinese Medicine
NA: Neuraminidase
mAb: Monoclonal antibody
FBS: Foetal bovine serum
DMEM: Dulbecco’s modified Eagle’s medium
FITC: Fluorescein isothiocyanate
