# Supplementary figures and images for: Antiviral effect of baicalin on Marek’s disease virus in CEF cells
Source: BMC Vet Res. 2020 Oct 2;16:371. doi: 10.1186/s12917-020-02595-x (PMC7532598; doi:10.1186/s12917-020-02595-x)

**Supplementary Figure 1**

The full-length blot images of Fig.2D in the manuscript.


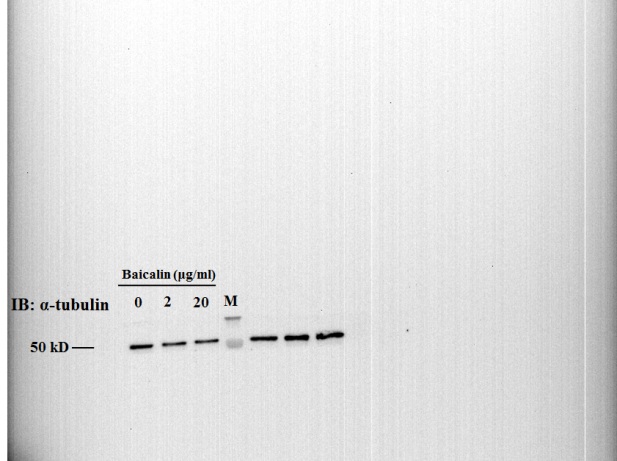

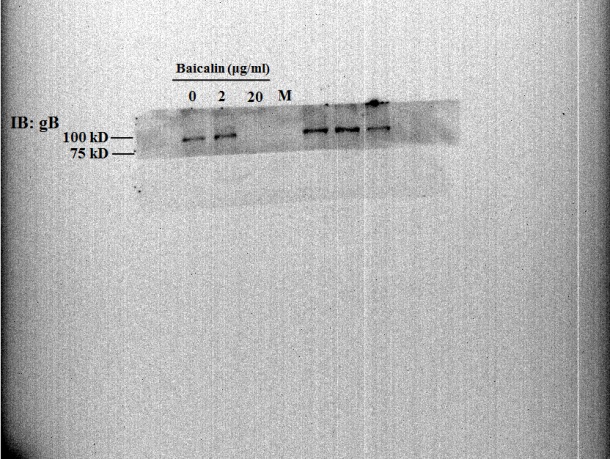

Supplement: Supplementary file 1 — Additional file 1 Figure S1. The full-length blot images of Fig.2d in the manuscript. [file 12917_2020_2595_MOESM1_ESM.docx]
